# Protein distribution affects muscle mass based on differences in postprandial muscle protein synthesis and plasma leucine in rats

**DOI:** 10.1186/1550-2783-9-S1-P23

**Published:** 2012-11-19

**Authors:** Layne E Norton, Gabriel J Wilson, Donald K Layman, Chris J Moulton, Peter J Garlick

**Affiliations:** 1 Division of Nutritional Sciences, Department of Food Science and Human Nutrition, University of Illinois at Urbana-Champaign, Urbana, IL, 61801, USA; 2 Department of Animal Sciences, University of Illinois at Urbana-Champaign, Urbana, IL, 61801, USA

## Background

Current protein recommendations are on a gram per day basis and do not account for individual meal responses of muscle protein metabolism. The purpose of this experiment was to examine if protein distribution could affect long-term body composition and muscle mass in rats isocaloric, isonitrogenous diets, using the same protein source.

## Methods

Male Sprague Dawley rats (275g) were fed isocaloric/isonitrogenous meals containing whey protein, with protein either distributed evenly at 16% of total energy over 3 meals (ED-Whey) or unevenly distributed over 3 meals (UD-Whey) with the first 2 meals containing only 8% of energy from whey protein and only the dinner meal containing sufficient protein to optimize muscle protein synthesis (MPS) (27.5% of total energy from whey protein) for 11 weeks. Measurements were taken to assess postprandial rates of MPS, plasma amino acids, mammalian target of rapamycin (mTOR) signaling, and the animals’ body composition was assessed by Dual energy X-ray absorptiometry (DXA). Hind limb muscle weights were taken to asses differences in muscle mass.

## Results

The ED-Whey treatment with evenly distributed protein produced a greater MPS response at the breakfast meal (p<0.05) and larger gastrocnemius muscle weights (p<0.05) compared to the UD-whey. While muscle mass was larger in the ED-Whey treatment at 11 weeks, total lean body mass was not different between groups. This may have been due to the large protein (i.e. nitrogen) content of the dinner meal in the UD-Whey group producing a shift in lean body mass deposition to the liver and visceral tissues, which were larger in the UD-Whey group.

## Conclusions

Muscle protein metabolism is regulated on a meal-to-meal basis and consuming multiple evenly distributed protein meals that stimulate MPS multiple times is superior for optimizing muscle mass compared to consuming the majority of protein at a single meal.

